# The Antioxidant Selenoprotein T Mimetic, PSELT, Induces Preconditioning-like Myocardial Protection by Relieving Endoplasmic-Reticulum Stress

**DOI:** 10.3390/antiox11030571

**Published:** 2022-03-17

**Authors:** Carmine Rocca, Anna De Bartolo, Maria Concetta Granieri, Vittoria Rago, Daniela Amelio, Flavia Falbo, Rocco Malivindi, Rosa Mazza, Maria Carmela Cerra, Loubna Boukhzar, Benjamin Lefranc, Jérôme Leprince, Youssef Anouar, Tommaso Angelone

**Affiliations:** 1 Laboratory of Cellular and Molecular Cardiovascular Pathophysiology, Department of Biology, Ecology and Earth Sciences (DiBEST), University of Calabria, 87036 Rende, Italy; anna.de_bartolo@unical.it (A.D.B.); mariaconcetta.granieri@unical.it (M.C.G.); flaviafalbo@gmail.com (F.F.); 2 UNIROUEN, Inserm U1239, Neuroendocrine, Endocrine and Germinal Differentiation and Communication (NorDiC), Rouen Normandie University, 76821 Mont-Saint-Aignan, France; loubna.boukhzar@univ-rouen.fr (L.B.); benjamin.lefranc@univ-rouen.fr (B.L.); jerome.leprince@univ-rouen.fr (J.L.); youssef.anouar@univ-rouen.fr (Y.A.); 3 Institute for Research and Innovation in Biomedicine, 76000 Rouen, France; 4 Department of Pharmacy, Health and Nutritional Sciences, University of Calabria, 87036 Rende, Italy; vittoria.rago@unical.it (V.R.); rocco.malivindi@unical.it (R.M.); 5 Laboratory of Organ and System Physiology, Department of Biology, Ecology and Earth Sciences (DiBEST), University of Calabria, 87036 Rende, Italy; daniela.amelio@unical.it (D.A.); rosa.mazza@unical.it (R.M.); maria_carmela.cerra@unical.it (M.C.C.); 6 National Institute of Cardiovascular Research (INRC), 40126 Bologna, Italy

**Keywords:** antioxidants, heart, ischemia/reperfusion injury, peptides, selenoproteins

## Abstract

Oxidative stress and endoplasmic reticulum stress (ERS) are strictly involved in myocardial ischemia/reperfusion (MI/R). Selenoprotein T (SELENOT), a vital thioredoxin-like selenoprotein, is crucial for ER homeostasis and cardiomyocyte differentiation and protection, likely acting as a redox-sensing protein during MI/R. Here, we designed a small peptide (PSELT), encompassing the redox site of SELENOT, and investigated whether its pre-conditioning cardioprotective effect resulted from modulating ERS during I/R. The Langendorff rat heart model was employed for hemodynamic analysis, while mechanistic studies were performed in perfused hearts and H9c2 cardiomyoblasts. PSELT improved the post-ischemic contractile recovery, reducing infarct size and LDH release with and without the ERS inducer tunicamycin (TM). Mechanistically, I/R and TM upregulated SELENOT expression, which was further enhanced by PSELT. PSELT also prevented the expression of the ERS markers CHOP and ATF6, reduced cardiac lipid peroxidation and protein oxidation, and increased SOD and catalase activities. An inert PSELT (I-PSELT) lacking selenocysteine was ineffective. In H9c2 cells, H_2_O_2_ decreased cell viability and SELENOT expression, while PSELT rescued protein levels protecting against cell death. In SELENOT-deficient H9c2 cells, H_2_O_2_ exacerbated cell death, that was partially mitigated by PSELT. Microscopy analysis revealed that a fluorescent form of PSELT was internalized into cardiomyocytes with a perinuclear distribution. *Conclusions:* The cell-permeable PSELT is able to induce pharmacological preconditioning cardioprotection by mitigating ERS and oxidative stress, and by regulating endogenous SELENOT.

## 1. Introduction

Cardiovascular diseases (CVDs) are still considered a major health problem worldwide, as well as the main cause of death in the Western world [1]. Coronary heart disease (CHD) is a major cardiac syndrome, the consequences of which are usually attributed to the detrimental effects induced by acute myocardial ischemia/reperfusion injury (MI/R); in patients presenting with acute myocardial infarction (AMI), the timely restoration of myocardial reperfusion, through thrombolytic therapy or primary percutaneous coronary intervention, represents the treatment of choice to reduce acute myocardial ischemic injury and limit infarct size [2]. However, reperfusion can paradoxically induce a specific additional component of irreversible injury exacerbating the necrosis and the final harmful effects to the myocardium and coronary microcirculation, ultimately leading to heart failure ([3] and references therein). Among the cardioprotective strategies against MI/R, numerous studies performed in animal and human models showed that ischemic preconditioning (IPC), consisting in heart exposure to brief, sublethal ischemic insults to render it more resistant against subsequent, prolonged ischemia, represents a powerful endogenous protective phenomenon ([4] and references therein). However, IPC cannot be applied in the case of an AMI since it is necessary to intervene before the onset of myocardial ischemia; thus, its clinical application remains limited. On the contrary, cardioprotection achieved by preconditioning-mimetic agents inducing pharmacological preconditioning (PPC) may have important implications in the clinical setting. IPC and PPC share underlying mechanistic pathways, and several agents including G protein-coupled receptor agonists, potassium channel openers, sodium/hydrogen exchange inhibitors, opioid receptor agonists and volatile anesthetics, have been tested for their preconditioning mimetic effects and therapeutic potential [5,6]. However, MI/R currently lacks an effective clinical therapy.

The contribution of oxygen free radicals and oxidative stress in the pathogenesis of MI/R is widely accepted. Reactive oxygen species (ROS), produced by diverse enzymes and organelles, including endoplasmic reticulum (ER), represent the initiators of MI/R damage and have been associated with necrosis, apoptosis, arrhythmogenesis and endothelial dysfunction following MI/R. ROS generated following I/R lead to a compromised post-ischemic myocardial recovery, thus representing a major cause of the reperfusion injury [7]. The increasing knowledge in the underlying mechanisms of oxidative stress during MI/R strongly supported the concept by which MI/R (as well as the major CVDs) induces ER stress (ERS) [8]. Under physiological conditions, ER is the main intracellular site responsible for biosynthesis, folding, modification and transport of proteins [9]. However, perturbations of the cellular redox regulation interfere with the conventional folding and translocation of secretory and membrane proteins within ER, triggering the unfolded protein response (UPR), eventually leading to ERS [10]. UPR is characterized by three major effector signaling pathways: inositol-requiring enzyme 1 (IRE1), PKR-like ER kinase (PERK), and activating transcription factor 6 (ATF6); they may deferentially or synergistically act and can serve both adaptive and maladaptive roles ([11] and references therein). In the context of the downstream effectors and target genes taking part to the complex ERS-activated UPR mechanism, if ER protein homeostasis is not restored, the excess and/or prolonged activation of UPR may induce apoptosis via induction of C/EBP homologous protein (CHOP), activation of Jun N-terminal kinase (JNK), and cleavage of caspase-12 ([11] and references therein).

In order to preserve their integrity against MI/R-induced oxidative stress, cells employ an arsenal of potent endogenous antioxidative systems, including selenoproteins, antioxidant selenium-containing proteins with different subcellular localization and chemical reactivities [12]. Selenoproteins contain selenocysteine (Sec) in their active site essential for catalytic activity and possess diverse biological functions in oxidoreductions, redox signalling, protein folding, Ca^2+^ homeostasis, apoptosis and cell survival, events that are altered in MI/R [12,13,14]. Diverse evidence reports that the dysfunction of selective selenoproteins can increase the susceptibility to oxidative stress and its related cardiovascular alterations, such as congestive heart failure and CHD [15]. Basic and clinical research are ongoing to decipher the mechanism underlying the action of crucial endogenous antioxidant mechanisms, which may contribute to identify a possible therapeutic targeting/application against oxidative stress-related CVDs. In this context, we and others have shown that selenoprotein T (SELENOT), which belongs to a group of ER-resident redoxins and the gene disruption of which develops a lethal embryonic phenotype in mice, exerting crucial oxidoreductase activity through its redox catalytic site, consisting in a CVSU amino acid sequence, where U represents a Sec residue ([16] and references therein). In particular, our recent findings suggest that SELENOT is required during early hyperplastic growth of cardiomyocytes and is essential for cardiomyocyte differentiation and protection, likely acting as a redox-sensing protein [17]. We also showed that SELENOT specific knockdown in corticotrope cells associates with UPR and ERS and impaired ER associated protein degradation (ERAD), suggesting its fundamental function in maintaining ER homeostasis [18].

The physiological impact of SELENOT during heart development, as well as its capacity to reduce free radical level and to prevent ERS prompted us to investigate whether SELENOT can play a role in preventing MI/R and ERS. Particularly, since the Sec residue of CVSU sequence of SELENOT can form a selenosulfide bridge with the upstream Cys residue and that this motif can interact with other cellular components via redox reactions, we designed a small peptide (PSELT), encompassing the redox active site of SELENOT, to explore: (1) its ability to induce ischemic preconditioning-like myocardial protective effects and its underlying mechanisms of cardioprotection in an isolated rat heart model of I/R insult; (2) its protective action against exposure to hydrogen peroxide (H_2_O_2_) in H9c2 cardiomyoblast cells; (3) the contribution of the endogenous SELENOT in the exogenous PSELT-dependent cell protection during H_2_O_2_ treatment; and (4) its ability to penetrate into the cardiomyocyte and its intracellular distribution.

## 2. Materials and Methods 

### 2.1. Peptides and Drugs

The peptides PSELT (H-Phe-Gln-Ile-Cys-Val-Ser-Sec-Gly-Tyr-Arg-OH), [Ser46,49] PSELT [called inert PSELT (I-PSELT) as a control], and PSELT-dansyl were synthesized by Fmoc solid phase methodology on an automated peptide synthesizer (CEM, Saclay, France) as previously described [19]. KCl, NaCl, NaHCO_3_, CaCl_2_, MgSO_4_, KH_2_PO_4_, NaH_2_PO_4_, Na_2_HPO_4_, mannitol, glucose, Na-pyruvate, β-nicotinamide adenine dinucleotide (NADH), reduced disodium salt hydrate, 2,4 dinitrophenylhydrazine (DNPH), 2-thiobarbituric acid (TBA), bovine serum albumin (BSA), butylated hydroxyanisole, diethyl ether, ethylenediaminetetraacetic acid (disodium salt), diethylenetriamine pentaacetic acid, pyrogallol, streptomycin sulfate, tween-20, hydrogen peroxide (H_2_O_2_), and urea were purchased from Sigma Aldrich (St. Louis, MO, USA). Tunicamycin (TM) was from Cayman Chemical (Ann Arbor, MI, USA), Dulbecco’s Modified Eagle Medium/Nutrient Mixture F-12 (DMEM/F-12), penicillin/streptomycin and fetal bovine serum (FBS) were provided by Thermo Fisher Scientific (Waltham, MA, USA). All the solutions were freshly prepared before starting the experiments. Absolute ethanol, ethyl acetate, hydrochloric acid, methanol, and trichloroacetic acid (TCA) were from Carlo Erba Reagents (Cornaredo, Milano, Italy). 

### 2.2. Animals 

Male Wistar rats (Envigo, Udine-Italy), weighing between 250 and 300 g, were housed, two per cage, under standard conditions of light and temperature and in a ventilated environment. The animals had access to food and water ad libitum. The experiments were conducted according to the Declaration of Helsinki, Italian law (D.L. 26/2014), the Guide for the Care and Use of Laboratory Animals published by the US National Institutes of Health (2011), and the Directive 2010/63/EU of the European Parliament on the protection of animals used for science. The scientific project was approved by the Italian Ministry of Health, Rome, and by the Ethics Review Board of the University of Calabria.

### 2.3. Langendorff Isolated Rat Heart Perfusion 

Rats were intraperitoneally injected with ethyl carbamate (2 g/kg body weight) for anesthetization and euthanized. Then, the hearts were connected to the Langendorff apparatus to start the retrograde perfusion, as reported in previous publications [17,20,21].

Hearts were perfused with Krebs–Henseleit (KH) solution (pH 7.4, gassed with 95% O_2_ and 5% CO_2_) containing in mM: 4.7 KCl, 113 NaCl, 25 NaHCO_3_, 1.8 CaCl_2_, 1.2 MgSO_4_, 1.2 KH_2_PO_4_, 1.1 mannitol, 11 glucose, and 5 Na-pyruvate. In order to continuously record the cardiac mechanical parameters and to allow isovolumic contractions, a water-filled latex balloon, connected to a pressure transducer (BLPR; WRI, Inc., Sarasota, FL, USA), was inserted through the mitral valve into the left ventricle (LV). The balloon was filled to obtain a LV end-diastolic pressure (LVEDP) of 5–8 mmHg during basal conditions. A second pressure transducer, located above the aorta, was employed for recording the coronary pressure (CP). Hemodynamic parameters were monitored and analyzed using a PowerLab data acquisition system (AD Instruments, Sydney, New South Wales, Australia) and the performance variables were measured every 10 min.

### 2.4. Experimental Protocols

#### *2.4.1. Ischemia/Reperfusion (I/R) Protocol* 

Hearts were allowed to stabilize for 40 min with KH solution during which the baseline parameters were recorded. After the stabilization period, the hearts underwent to 30 min of global, no-flow ischemia followed by 120 min of reperfusion to reproduce I/R injury. Cardiac performance was evaluated in pre- and post-ischemic phases by assessing developed LV pressure (dLVP) recovery, an index of contractile activity, and LVEDP, an index of contracture, defined as an increase in this parameter of 4 mmHg above the baseline level [17,20,22].

#### *2.4.2. Experimental Groups* 

Hearts were randomly assigned to one of the following experimental groups:

(a). Sham group: hearts underwent only 190 min of perfusion;
(b). I/R Control group: hearts were exposed to I/R protocol;
(c). PSELT group, pre-conditioning with PSELT: after stabilization, PSELT was infused for 20 min before I/R at the concentration of 5 nM according to our prior ex vivo study [17], where PSELT elicited cardioprotection as post-conditioning agent;
(d). TM group, pre-conditioning with tunicamycin (TM): after stabilization, TM (2.5 µg/mL) was infused for 5 min before I/R [23];
(e). TM + PSELT group, pre-conditioning with TM + PSELT: after stabilization, TM was infused for 5 min followed by a 20 min 5 nM PSELT infusion before I/R;
(f). I-PSELT group, pre-conditioning with inert PSELT (I-PSELT): after stabilization, 5 nM I-PSELT was infused for 20 min before I/R [17].

### 2.5. Assessment of Myocardial Injury

#### *2.5.1. Lactate Dehydrogenase Activity in Coronary Effluent* 

The coronary effluent was collected in ice-cold tubes immediately before ischemia and at 10, 20, and 30 min of reperfusion for the assessment of LDH activity [24], that was evaluated spectrophotometrically by a Multiskan™ SkyHigh (Thermo Fisher Scientific Inc., Waltham, MA, USA) according to the method of McQueen [25] and as previously reported [20]. LDH activity, expressed as IU/mL, was obtained by monitoring the decrease in absorbance at 340 nm, resulting from the oxidation of NADH.

#### *2.5.2. Infarct Size* 

At the end of I/R protocols, the hearts were quickly removed from the Langendorff apparatus and infarct areas were measured by nitro blue tetrazolium staining, as previously described [20,22]. The analysis was performed in a blinded fashion by an independent observer who was not aware of the type of the intervention and unstained necrotic tissue was carefully separated from stained viable tissue. The infarct size (IS) was expressed as a percentage of the total LV mass [20,22].

### 2.6. Biochemical Analyses

After I/R protocols, one part of the LV of each heart was processed for protein immunoblotting assays, while the other part was used for evaluating antioxidant enzyme activities, lipid peroxidation and protein oxidation. 

#### *2.6.1. Superoxide Dismutase (SOD) Assay* 

SOD activity was measured by following the inhibition of pyrogallol autooxidation according to the method of Marklund and Marklund (1974) [26]. In brief, a buffer with 50 mM Tris HCl, pH 8.2 containing 1 mM diethylenetriamine pentaacetic acid was used to homogenize LV tissues, that were then centrifuged for 20 min at 20,000× *g*. Supernatant was then employed for the enzyme activity determination. The reaction was initiated by adding 0.2 mM pyrogallol and its autooxidation was monitored by measuring absorbance at 420 nm for 3 min using a Multiskan™ SkyHigh (Thermo Fisher Scientific Inc., Waltham, MA, USA). The enzyme activity was assessed as units per milligram of protein, where 1 U of SOD corresponds to the amount of enzyme able to inhibit the rate of pyrogallol autoxidation by 50%. 

#### *2.6.2. Catalase (CAT) Assay* 

LV of I/R-exposed hearts of each experimental group was homogenized in a buffer containing 50 mM potassium phosphate buffer at pH 7.4 (weight-to-volume ratio of 1:10). After centrifugation at 20,000× *g* for 30 min, supernatant was collected and used for CAT activity assay according to the method of Clairborne (1985) [27]. CAT was estimated by measuring the change in the absorbance for H_2_O_2_ consumption at 240 nm for 3 min. One unit of CAT activity reflects the micromoles of H_2_O_2_ decomposed per min using the molar extinction coefficient of H_2_O_2_ (43.6 M^−1^ cm^−1^). Specific activity of the enzyme was expressed in μmoles/mg protein.

#### *2.6.3. Thiobarbituric Acid Reactive Substances (TBARS) Assay* 

The lipid peroxidation was determined by thiobarbituric acid reactive substances (TBARS) assay, as previously described [28]. Briefly, LV of hearts exposed to I/R protocol was homogenized in 0.9% KCl (pH 7.4) (10% *w*/*v*) and after homogenization samples were incubated at 37 °C. Then, 1 mL of 40% (*w*/*v*) TCA and 1 mL of 0.2% (*w*/*v*) TBA were added to 2 mL of each cardiac homogenate. 2% (*w*/*v*) butylated hydroxytoluene was also added to the TBA reagent mixture for preventing artificial lipid peroxidation during the procedure [29]. The mix was incubated at 100 °C for 15 min, brought to room temperature and 2 mL of 70% (*w*/*v*) TCA was added. The samples were then centrifuged (3500 rpm for 20 min) and the color intensity, determined spectrophotometrically at 523 nm, was interpreted as TBARS levels and expressed in nmoles/g heart tissue.

#### *2.6.4. Protein Carbonyl Content Assay* 

For protein carbonyl content determination, (i.e., an indicator of protein damage following oxidative stress), LV samples of I/R-exposed hearts were homogenized in 50 mM KH_2_PO_4_, 1 mM EDTA buffer pH = 7.4. Protein oxidation was evaluated by the derivatization of protein carbonyl groups with DNPH according to the method of Reznick and Packer (1994) [30]. After centrifuging cardiac homogenate at 10,000× *g* for 15 min, their supernatants were incubated at room temperature for other 15 min with 1% streptomycin to remove nucleic acids, which may erroneously overestimate carbonyls, and centrifuged at 6000× *g* for 10 min at 4 °C. The resulting supernatant was used for the subsequent assay made as previously reported [31,32]. The protein carbonyl concentration was calculated by measuring the absorbance at 375 nm, using the extinction coefficient for DNPH (22 mM^−1^ cm^−1^) and was expressed in nmol/mg protein.

### 2.7. Western Blot Analyses on Cardiac Tissues

Immunoblotting procedures were carried out on heart tissue extracts, as reported in our previous publications [17,20,22,33]. Forty micrograms of protein, the concentration of which was determined using the Bradford assay (Sigma-Aldrich, St. Louis, MO, USA), were loaded on 10% (for ATF-6α, sc-166659, Santa Cruz Biotechnology, Dallas, TX, USA) and 12% (for SELENOT, rabbit polyclonal antibody, LS-C168948, LSBio|LifeSpan BioSciences, Seattle, WA and CHOP, sc-7351, Santa Cruz Biotechnology) sodium dodecyl sulphate–polyacrylamide gel electrophoresis (SDS–PAGE) gels, separated and electrophoretically transferred to polyvinyl difluoride (PVDF) membranes (GE Healthcare, Chicago, IL, USA). After blocking non-specific protein-binding sites with 5% (*w*/*v*) non-fat milk in Tris-buffered saline 0.1% (*w*/*v*) Tween-20 for 1 h at room temperature, the PVDF membranes were incubated overnight at 4 °C with antibodies specific for the above antigens diluted 1:500 (ATF-6α and CHOP) and 1:1000 (SELENOT and β-actin) in Tris-buffered saline containing 0.1% Tween 20 and 5% nonfat dry milk. Antibody against β-actin (sc-81178, Santa Cruz Biotechnology) was used as loading control. The bound primary antibodies were detected using goat anti-rabbit (G-21234, Thermo Fisher Scientific, Waltham, MA, USA) or goat anti-mouse (G-21040, Thermo Fisher Scientific) horseradish peroxidase-conjugated secondary antibody diluted 1:2000 in Tris-buffered saline containing 0.1% Tween 20 and 5% nonfat dry milk for 2 h at room temperature and an enhanced chemiluminescence (ECL) Western blotting detection system (Amersham, IL, USA). Densitometric analysis of the bands was performed after digitalization by evaluating the areas and the pixel intensity and subtracting the background. The analyses were conducted using ImageJ 1.6 (National Institutes of Health, Bethesda, MD, USA). 

### 2.8. Cell Culture and Treatments

The H9c2 rat cardiomyoblast cell line was obtained from the American Type Culture Collection (ATCC) (Manassas, VA, USA) (Cat# CRL-1446) and cultured in Dulbecco’s Modified Eagle Medium/Nutrient Mixture F-12 (DMEM/F-12, Gibco, Thermo Fisher Scientific, Waltham, MA, USA) supplemented with 10% fetal bovine serum (FBS, Gibco) and 1% penicillin/streptomycin (Thermo Fisher Scientific, Waltham, MA, USA). Cells were then incubated at 37 °C in humidified atmosphere with 5% CO_2_. After reaching ~80% of density in 100-mm dishes, cells digestion was performed using 0.25% Trypsin-EDTA (1X) (Gibco) according to a 1:2 ratio, following manufacturer’s instructions (ATCC). To perform experiments, cells were seeded and incubated for 48 h at 37 °C, 95% O_2_ and 5% CO_2_, as previously reported [34,35,36,37].

#### *2.8.1. Cell Viability by 3-(4,5-Dimethylthiazol-)2,5-diphenyl Tetrazolium Bromide (MTT) Assay* 

MTT assay was performed to assess H9c2 cell viability. To this aim, cells were seeded at a density of 5 × 10^3^ cells/well in 96-well plate and then treated with PSELT (from 5 to 1000 nM) or inert PSELT (I-PSELT) (from 5 to 1000 nM) for 24 h except for control cells that were treated with vehicle (UltraPure RNase/DNase-free distilled water). Then, H9c2 cardiomyoblast cells were treated with 200 μM H_2_O_2_ for 3 h, with or without PSELT or I-PSELT, to establish an oxidative stress model, as previously reported [38]. At the end of treatments, the cell culture medium was replaced with 100 µL of 2 mg/mL MTT solution (Sigma Aldrich, St. Louis, MO, USA) and cells were incubated for 4 h at 37 °C, 5% CO_2_. Finally, the MTT solution was removed and the formazan crystals were solubilized in DMSO for 30 min. The absorbance was recorded at 570 nm (Multiskan™ SkyHigh (Thermo Fisher Scientific Inc., Waltham, MA, USA). 

The percentage of cell viability, reported as percentage of cells survival relative to control cells, derives from the means of absorbance values of six wells in each experimental group. The experiment was repeated three independent times [33,34,35].

#### *2.8.2. Western Blot Analysis on H9c2 Cells* 

Following the specific treatments with vehicle, H_2_O_2_ (200 µM) and H_2_O_2_ + PSELT (from 5 to 1000 nM), total proteins of H9c2 cells were extracted using RIPA lysis buffer (Sigma Aldrich) appropriately supplemented with a mixture of protease inhibitors [(aprotinin 20 μg/mL, phenylmethylsulfonyl fluoride 1%, sodium orthovanadate 2 μM (Sigma-Aldrich)]. Scraped cells were transferred in microtubes and incubated on ice for 30 min with intermittent mixing; then, they were centrifuged at 12,000× *g* for 15 min at 4 °C. The concentration of proteins in the supernatant was assessed by Bradford assay. Then, 30 micrograms of protein were loaded on 12% SDS-PAGE gel (anti-SELENOT). The next steps were performed as described in the section above. 

#### *2.8.3. Short Interfering RNA (siRNA) Transfection for SELENOT Silencing* 

H9c2 cardiomyoblasts (1 × 10^5^ cells/well) were seeded in 6-well plates 48 h before transfection. For gene silencing, siRNA (100 nM) against SELENOT was transfected into H9c2 cardiomyocytes using Lipofectamine 2000 transfection reagent following the manufacturer’s instructions (Invitrogen, Thermo Fisher Scientific, Waltham, MA, USA). Scrambled siRNA used as control and siRNA for SELENOT were obtained from Santa Cruz Biotechnology. Six hours post-transfection, serum-free medium was replaced with growth medium and cells were incubated for 36 h at 37 °C, 5% CO_2_. The efficiency of knockdown was assessed by Western blot analysis. Afterwards, H9c2 cells were seeded at a density of 5 × 10^3^ cells/well in 96-well plate and then transfected with SELENOT siRNA (si-SELENOT) or si-RNA negative control (si-NC) as above reported. After transfection, H9c2 cardiomyocytes were pre-treated with increasing concentrations of PSELT (from 5 to 1000 nM) for 24 h and then exposed to H_2_O_2_ (200 μM) for additional 3 h. At the end of treatments, H9c2 cell viability was evaluated by MTT assay as previously reported.

The percentage of cell viability, reported as percentage of cells survival relative to si-RNA negative control cells, derives from the means of absorbance values of six wells in each experimental group. The experiment was repeated three independent times.

#### *2.8.4. Immunofluorescence Analysis* 

H9c2 cells were seeded in chambers with coverslip (5 × 10^4^ cells per chamber), incubated for 48 h at 37 °C, 5% CO_2_ and then treated with vehicle or PSELT-dansyl 30 nM for 15 min. Cells were washed with DPBS and fixed for 10 min with ice-cold methanol. Fixed cells were rinsed with DPBS two times and permeabilization phase was performed using 0.1 % Triton X-100 in DPBS for 30 min at RT. Permeabilized H9c2 cells were then washed with DPBS and blocked with 1% BSA in DPBS for 30 min at RT. For immunofluorescence analysis, cells were incubated with a primary antibody against laminin (diluted 1:100) overnight at 4 °C and then stained with a donkey anti-rabbit secondary antibody, Alexa Fluor 488 (diluted 1:400) for 1 h at RT. The cells were then washed twice with DPBS and incubated with propidium iodide (PI) for nuclei staining. Images were obtained using an Olympus Fluoview FV3000 microscope and the images were taken with FV31S-SW software. Quantification of fluorescence signals was carried out using CellSens Dimension software 1.7 (Count&Measure Full, Olympus Europa SE & Co., Hamburg, Germany).

### 2.9. Statistical Analyses

Experimental data were expressed as means ± SEM. A two-way ANOVA and non-parametric Bonferroni’s multiple comparison test (for post-ANOVA comparisons) were used for hemodynamic analyses and LDH evaluation. A one-way ANOVA and the non-parametric Newman–Keuls multiple comparison test (for post-ANOVA comparisons), one-way ANOVA followed by Dunnett’s multiple comparison test, and unpaired *t*-test were used for the other analyses when appropriate. Values (* *p* ≤ 0.05, ** *p* ≤ 0.01, *** *p* ≤ 0.001) were statistically significant. The statistical analysis was conducted using Prism 5 (GraphPad Software, La Jolla, CA, USA).

## 3. Results 

### 3.1. Action of PSELT on Post-Ischemic Systolic and Diastolic Recovery 

To determine the potential cardioprotective effect of PSELT on I/R injury, we employed an ex vivo Langendorff model in the presence or absence of the ER stress inducer TM. In order to evaluate the endurance and the stability of the preparation, the variables of the cardiac performance were recorded every 10 min indicating that the heart is stable up to 190 min. Indeed, the cardiac parameters of sham hearts at the end of perfusion were: dLVP = 65 ± 3 mmHg; LVEDP = 5 ± 0.6 mmHg; CP = 68 ± 1.1 mmHg; HR = 250 ± 8 mmHg.

While, considering all experimental groups, the cardiac parameters obtained after 40 min of stabilization, before I/R protocols, were: dLVP = 68 ± 2 mmHg; LVEDP = 5 ± 0.4 mmHg; CP = 66 ± 0.6 mmHg; HR = 260 ± 2 mmHg. Performance variables were measured every 10 min. 

**Figure 1:**
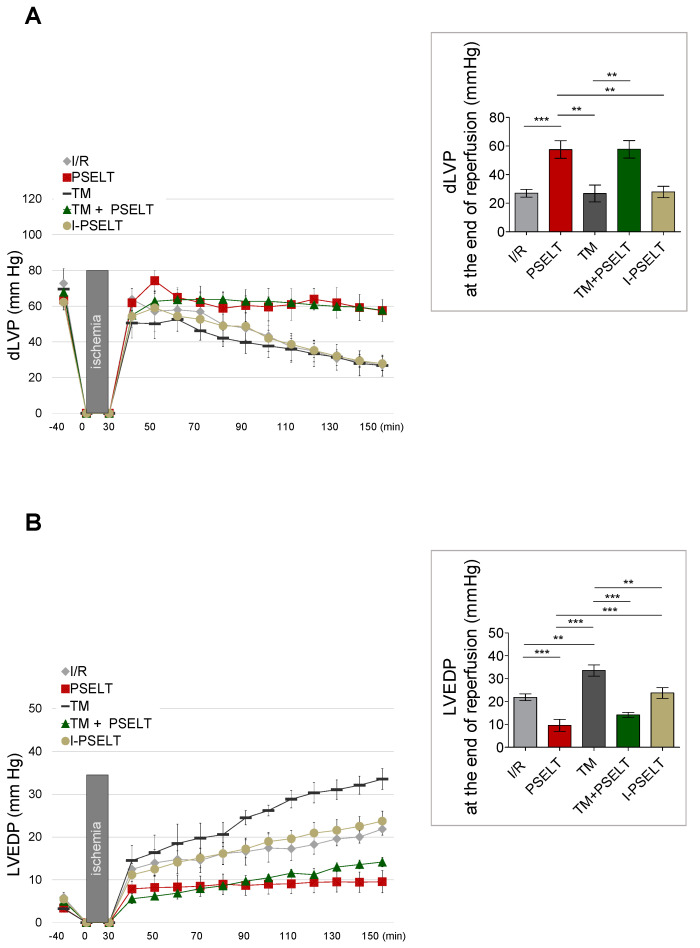
**Effect of the selenoprotein T-derived peptide 43-52 (PSELT) and tunicamycin (TM), alone and in combination, on systolic and diastolic functions of Langendorff-perfused hearts subjected to I/R injury**. (**A**) dLVP and (**B**) LVEDP variations. Black boxes indicate ischemic administration (Bonferroni multiple comparison test). dLVP = 6.97 % of total variation between groups (*p* < 0.001); LVEDP = 20.70 % of total variation between groups (*p* < 0.001). dLVP and LVEDP values at the end of reperfusion are showed in the inset graph. Data are expressed as changes of dLVP and LVEDP values (millimeters of mercury) from stabilization to the end of the 120 min of reperfusion with respect to the baseline values for control (I/R alone), PSELT, TM, TM + PSELT (n = 6 hearts/group) and I-PSELT groups (n = 4 hearts). *p* < 0.01 (**), *p* < 0.001 (***) (one-way ANOVA and Newman–Keuls multiple comparison test).

The pre-conditioning (PreC) cardioprotective action of PSELT on the post-ischemic systolic and diastolic function was examined by analyzing the developed LVP recovery (dLVP; i.e., the inotropic activity) and the LVEDP value (i.e., the contracture state) before and after ischemia. As depicted in Figure 1A, showing dLVP values during the reperfusion and at the end of reperfusion, in the control I/R and TM groups, the dLVP was significantly lower than before ischemia (I/R group: dLVP: 27 ± 3 mmHg at the end of reperfusion; values at baseline: 73 ± 8 mmHg; TM group: dLVP: 27 ± 6 mmHg at the end of reperfusion; baseline values: 70 ± 4 mmHg); in contrast, in PSELT and TM + PSELT groups, where PSELT was administered at the concentration of 5 nM before I/R [17], dLVP recovery significantly improved during reperfusion (PSELT group: dLVP: 58 ± 6 mmHg at the end of reperfusion; baseline values: 64 ± 6 mmHg; TM + PSELT group: dLVP: 58 ± 6 mmHg at the end of reperfusion; baseline values: 68 ± 4 mmHg) (Figure 1A). Conversely, I-PSELT (5 nM) showed no action, since in I-PSELT group dLVP was not significantly improved in the post-ischemic phase compared to the pre-ischemic phase (I-PSELT group: dLVP: 28 ± 4 mmHg at the end of reperfusion; baseline values: 62 ± 3 mmHg) (Figure 1A).

LVEDP (i.e., cardiac contracture state) is considered an index of the diastolic function in the post-ischemic phase (i.e., LVEDP 4 mmHg or more above baseline level) [17,20,22]. Our results showed that in I/R control group, LVEDP significantly increased during reperfusion [I/R group: LVEDP: 22 ± 1 mmHg at the end of reperfusion; baseline values: 6 ± 1 mmHg], in TM group, the LVEDP increase was more pronounced since it significantly increased compared with I/R group (TM group: LVEDP: 34 ± 2 mmHg at the end of reperfusion; baseline values: 3 ± 1 mmHg), attesting an additive effect of TM-induced ER stress to I/R protocol, whereas in the PSELT and TM + PSELT groups, this parameter was significantly lower compared to I/R and TM alone, respectively (PSELT group: LVEDP: 10 ± 3 mmHg at the end of reperfusion; baseline values: 4 ± 1 mmHg; TM + PSELT group: LVEDP: 14 ± 1 mmHg at the end of reperfusion; baseline values: 5 ± 1 mmHg) (Figure 1B). In addition, in I-PSELT group, the cardiac contracture was not abolished, since LVEDP significantly augmented during reperfusion (I-PSELT group: LVEDP: 24 ± 2 mmHg at the end of reperfusion; baseline values: 5 ± 1 mmHg) (Figure 1B). 

### 3.2. Effect of PSELT on Infarct Size (IS) and Lactate Dehydrogenase Release (LDH)

The cardioprotective effect of PSELT as PreC agent was also evaluated by measuring the extent of myocardial infarction (ischemia-induced LDH release and IS, in the presence or absence of TM).

**Figure 2:**
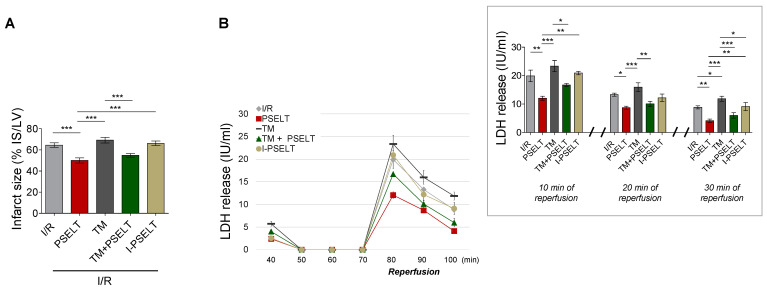
**Effect of PSELT and TM, alone and in combination, on infarct size and LDH activity in coronary effluent of Langendorff-perfused hearts subjected to I/R injury**. (**A**) Infarct size (IS) for I/R, PSELT, TM, TM + PSELT (n = 6 hearts/group) and I-PSELT groups (n = 4 hearts). The amount of necrotic tissue measured after 30 min global ischemia and 120 min reperfusion is expressed as a percentage of the LV mass (%IS/LV). *p* < 0.05 (*), *p* < 0.01 (**), *p* < 0.001 (***) (one-way ANOVA and Newman–Keuls multiple comparison test). (**B**) LDH activity was determined in coronary effluent 5 min before ischemia and 10, 20 and 30 min after ischemia in the reperfusion phase. LDH variations for I/R, PSELT, TM, TM + PSELT (n = 6/group) and I-PSELT groups (n = 4) (Bonferroni multiple comparison test). A total of 3.26 % of total variation between groups (*p* < 0.001). Inset graph shows LDH activity at the end of 10, 20 and 30 min of reperfusion. Data are expressed as IU/mL for I/R, PSELT, TM, TM + PSELT (n = 6/group) and I-PSELT groups (n = 4). *p* < 0.05 (*), *p* < 0.01 (**), *p* < 0.001 (***) (one-way ANOVA and Newman–Keuls multiple comparison test).

IS (expressed as a percentage of LV mass) values were similar in the I/R control and TM groups (~65 ± 2% and ~69 ± 3% of IS/LV, respectively) (Figure 2A). In contrast, IS was significantly reduced in the presence of PSELT compared with I/R and TM alone groups, being ~50 ± 2% in PSELT group and ~55 ± 2% in TM + PSELT group (Figure 2A). I-PSELT was not able to reduce IS compared to I/R control group; indeed, the infarct area in I-PSELT group was ~66 ± 2% (Figure 2A).

The severity of cardiac cellular damage was assessed by evaluating the activity of LDH enzyme from coronary effluents (collected during the first 10, 20 and 30 min of reperfusion) [24]. Figure 2B, representing LDH release during the first 30 min of reperfusion and at 10, 20, and 30 min of reperfusion, shows that the post-ischemic LDH release was markedly increased compared with pre-ischemic phase in I/R control and TM groups (Figure 2B), while PSELT significantly attenuated LDH release in coronary effluent during reperfusion, both in the absence and presence of TM (PSELT and TM + PSELT groups) with respect to I/R and TM alone groups, respectively (Figure 2B). The enzyme activity in I-PSELT-treated hearts was similar to that of I/R control hearts. 

### 3.3. Effect of PSELT on Cardiac Oxidative Stress and the Activity of Endogenous Antioxidant Enzymes 

**Figure 3:**
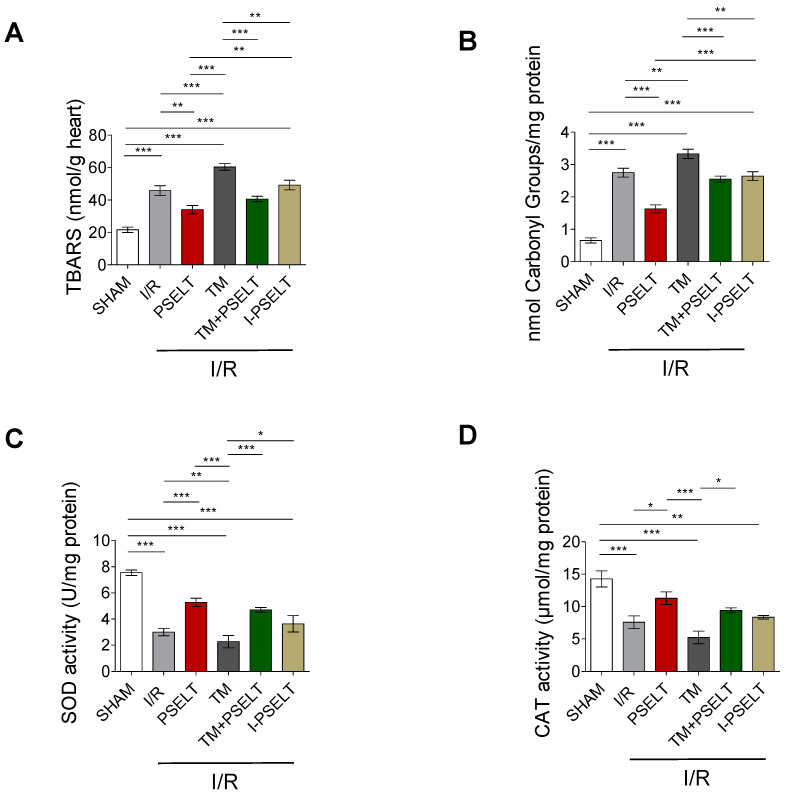
**Effect of PSELT and TM, alone and in combination, on lipid peroxidation, protein oxidation, and antioxidant enzymes**. (**A**) TBARS levels, (**B**) protein carbonyl groups, (**C**) SOD enzyme activity and (**D**) CAT enzyme activity in sham and I/R-exposed hearts for I/R alone, PSELT, TM, TM + PSELT (n = 6/group) and I-PSELT groups (n = 4). *p* < 0.05 (*), *p* < 0.01 (**), *p* < 0.001 (***) (one-way ANOVA and Newman–Keuls multiple comparison test).

In order to determine the potential ability of PSELT to reduce cardiac oxidative stress induced by I/R maneuver, with or without TM, selective oxidative stress-related markers and the activity of the endogenous antioxidant enzymes SOD and CAT were measured. Figure 3A shows that the levels of TBARS, a lipid peroxidation indicator, increased in I/R exposed hearts compared with sham hearts, whereas PSELT significantly attenuated cardiac TBARS formation with respect to I/R control group; similarly, in TM group, TBARS levels were significantly higher than those of sham and I/R groups, while in TM + PSELT group, they significantly decreased compared to TM alone group (Figure 3A). Similar to I/R group, in I-PSELT-treated hearts, TBARS levels were significantly higher than those of sham hearts (Figure 3A). A similar trend was observed for the estimation of protein carbonyl groups, used as a marker of protein damage following oxidative stress (Figure 3B).

Regarding the activity of endogenous antioxidant enzymes, I/R and TM hearts exhibited a significantly decrease of both SOD and CAT activity respect to sham hearts (Figure 3C,D). Conversely, in the PSELT and TM + PSELT groups, SOD and CAT activity were significantly higher than in the I/R and TM groups, respectively (Figure 3C,D). I-PSELT treatment was not able to prevent the decrease of SOD and CAT activity induced by I/R, since in I-PSELT group the levels of these enzymes were similar to those of I/R control group (Figure 3C,D).

### 3.4. Influence of PSELT on the Expression of SELENOT and ER Stress Response Markers in I/R Hearts

**Figure 4:**
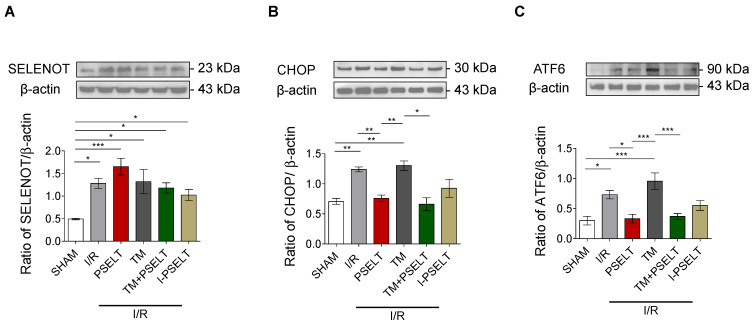
**Effect of PSELT and TM, alone and in combination on protein expression of SELENOT and ERS-related markers.** Western blot analysis of (**A**) SELENOT, (**B**) CHOP, and (**C**) ATF6 in sham and I/R-exposed hearts for I/R alone, PSELT, TM, TM + PSELT and I-PSELT groups (n = 4 hearts/group). Histograms represent the ratio of densitometric analysis of protein/loading control. *p* < 0.05 (*), *p* < 0.01 (**), *p* < 0.001 (***) (one-way ANOVA and Newman–Keuls multiple comparison test).

To evaluate whether PSELT could affect the cardiac expression of SELENOT and selective ER stress response markers, we performed western blot analyses on the LV extracts deriving from the hearts exposed to I/R protocols of each experimental group. Our results showed first that after I/R protocols, SELENOT expression levels significantly increased compared with sham hearts (Figure 4A). Moreover, PSELT treatment further enhanced the protein expression (Figure 4A). Similarly, in TM, TM + PSELT and I-PSELT groups, SELENOT protein expression was significantly higher than sham group (Figure 4A). We then examined whether PSELT could influence the cardiac expression levels of CHOP, a hallmark of UPR and ATF6, a sensor of ER stress. Western blot and densitometric analyses showed in Figure 4B indicate that I/R, triggered a significant increase of cardiac CHOP expression both in presence and absence of TM and that PSELT was able to significantly reduce the protein expression in both PSELT and TM + PSELT groups (Figure 4B). A similar trend was observed for ATF6 expression (Figure 4C). In addition, in this case, I/R and TM-treated hearts exhibited a significant increase of ATF6 expression, that was mitigated in the hearts preconditioned with PSELT, while I-PSELT was unable to significantly reduce the protein expression compared with I/R alone group (Figure 4C).

### 3.5. Effect of PSELT against Oxidative Stress in H9c2 Cells

**Figure 5:**
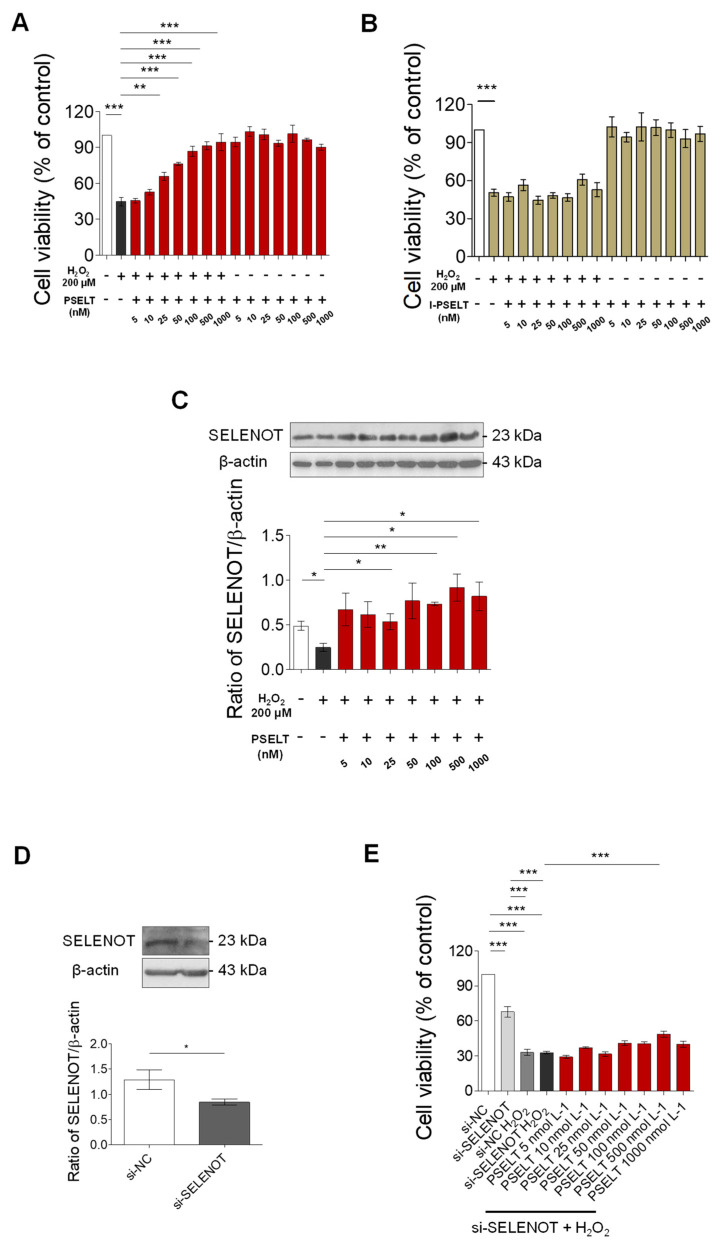
**Effects of PSELT against oxidative stress-induced damage in H9c2 cells.** (**A**) Effect of PSELT on cell viability in H9c2 cells exposed to a pre-treatment with increasing concentration of PSELT (5, 10, 25, 50, 100, 500, and 1000 nM) or (**B**) inert PSELT (I-PSELT) (5, 10, 25, 50, 100, 500, and 1000 nM) for 24 h and then exposed to H_2_O_2_ (200 μM) for additional 3 h. Cell viability was assessed using MTT assay. Results are represented as mean ± SEM (n = 6 per group). Significant differences were detected by one-way ANOVA followed by Newman–Keuls multiple comparison test, *p* < 0.05 (*), *p* < 0.01 (**), *p* < 0.001 (***). (**C**) Western blot analysis of SELENOT in H9c2 cells exposed to vehicle (indicated as control) or pre-treated with increasing concentration of PSELT (5, 10, 25, 50, 100, 500, and 1000 nM) for 24 h and then exposed to H_2_O_2_ (n = 3 independent experiments) (rabbit polyclonal antibody against SELENOT and β-actin. Histograms represent the ratio of densitometric analysis of protein:loading control. Significant differences were detected by *t*-test. *p* < 0.05 (*), *p* < 0.01 (**). (**D**) Representative image of SELENOT knockdown in H9c2 cells, transfected with 100 nM siRNA-SELENOT or negative control (siRNA-NC). Data are shown as mean ± SEM from three independent experiments. Histograms represent the ratio of densitometric analysis of protein:loading control. Significant differences were detected by *t*-test. *p* < 0.05 (*) vs. si-NC group. (**E**) Effect of si-SELENOT gene silencing on cell viability in H9c2 cells pre-treated with increasing concentration of PSELT for 24 h and exposed to H_2_O_2_ for 3 h. Cell viability was evaluated using MTT assay and was expressed as the percentage of control cells only transfected with siRNA negative control (indicated as si-NC). Results are represented as mean ± SEM (n = 6 per group). Significant differences were detected by one-way ANOVA followed by Newman–Keuls multiple comparison test. *p* < 0.001 (***).

To test the protective effect of PSELT against a direct cardiac oxidative damage, we exposed H9c2 cardiomyoblasts to H_2_O_2_ to reproduce an in vitro model of oxidative stress. Our results indicate that the viability of H9c2 cells treated with H_2_O_2_ decreased compared with the control group (vehicle) (Figure 5A), while the viability of cells pre-treated with PSELT (5, 10, 25, 50, 100, 500, and 1000 nM) for 24 h before exposure to H_2_O_2_ significantly increased, in a concentration-dependent manner (EC_50_ value: ~30 nM), compared with H_2_O_2_ alone group (Figure 5A). The graph in Figure 5A also shows that PSELT alone, without H_2_O_2_, did not affect cell viability at any tested concentration. Figure 5B indicates that, in the same experimental conditions, I-PSELT was inactive, since no significant effects have been observed on viability of H9c2 cells at the concentration range of I-PSELT 5–1000 nM, in the presence of H_2_O_2_ compared with H_2_O_2_ alone group.

### 3.6. Role of Endogenous SELENOT in PSELT-Dependent H9c2 Cell Protection following H_2_O_2_ Exposure

To determine the possible contribution of endogenous SELENOT in PSELT-dependent cytoprotection in H9c2 cells during H_2_O_2_ exposure, we first evaluated the influence of PSELT on SELENOT expression in H9c2 cells exposed to H_2_O_2_. The results showed that H_2_O_2_ significantly decreased SELENOT expression levels with respect to control cells (Figure 5C), while PSELT treatment was able to rescue the protein levels during H_2_O_2_ treatment, as evidenced by its significant increase in H9c2 cells pre-treated with increasing concentrations (25, 100, 500, and 1000 nM) of PSELT before incubation with H_2_O_2_ (Figure 5C). Subsequently, we evaluated the effect of SELENOT knockdown by a SELENOT small interfering RNA (siRNA) on PSELT-induced cytoprotection in H9c2 cells during H_2_O_2_ exposure. To this aim, we firstly assessed the effectiveness of SELENOT knockdown in H9c2 cells with siRNA SELENOT (si-SELENOT). Western blot and densitometric analysis presented in Figure 5D indicate that the SELENOT expression levels were significantly decreased in si-SELENOT group compared with control group. Then, si-SELENOT transfected cells were pre-treated with PSELT (5, 10, 25, 50, 100, 500, and 1000 nM) for 24 h before H_2_O_2_ incubation. Results presented in Figure 5E indicate that si-SELENOT provoked a significant decrease of cell viability compared with control cells (si-NC) and that the treatment with H_2_O_2_, with or without si-SELENOT, further decreased cell viability with respect to si-NC control group. The figure also shows that PSELT (5, 10, 25, 50, 100, 500, and 1000 nM) administration in si-SELENOT transfected H9c2 cells exposed to H_2_O_2_ partially recovered cell viability, although only 500 nM concentration was statistically significant, compared with si-SELENOT H9c2 cells treated with H_2_O_2_.

### 3.7. Evaluation of Cardiomyocyte-Penetrating Capacity and Cell Distribution of PSELT 

**Figure 6:**
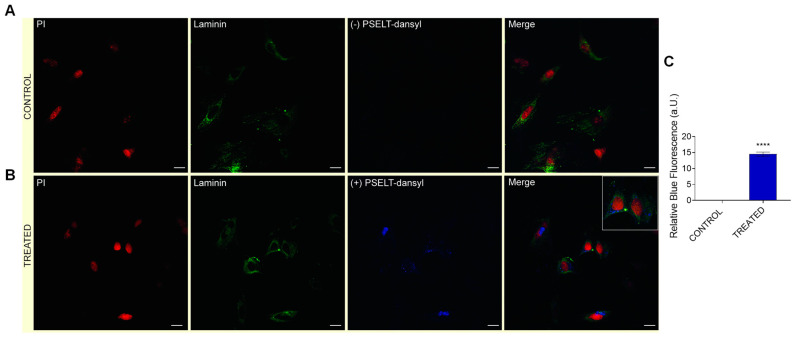
**Evaluation of PSELT ability to penetrate into the cardiomyocyte and its intracellular distribution.** Representative confocal microscopic images of H9c2 cardiomyocytes incubated for 15 min with (**A**) vehicle or (**B**) PSELT-dansyl (blue) 30 nM. Laminin antibody (Sigma Aldrich) was used as plasma membrane marker (green) and propidium iodide was used for nuclei staining (red). *Scale bars*: 12.5 μm. (**C**) Quantification of relative immunofluorescence of PSELT-dansyl in control and treated groups. Data are expressed as mean ± SEM [unpaired *t*-test, **** *p* < 0.001 (n = 4 per group)].

To establish whether PSELT could penetrate within the cell and study its distribution, we used PSELT-dansyl, consisting in a fluorescent form of PSELT obtained by coupling a dansyl fluorophore to the N-terminus of the peptide. For this purpose, H9c2 cells were incubated for 15 min with vehicle or PSELT-dansyl. Confocal microscopy analysis revealed that the peptide was internalized into cardiomyocytes showing a perinuclear distribution, as evinced by PSELT-dansyl fluorescent dots (Figure 6).

## 4. Discussion 

Ischemic heart disease and the consequent heart failure that often occurs still represent the major causes of death and disability in the world; therefore, novel therapeutic strategies are urgently needed to protect the heart against the detrimental effects induced by acute MI/R and improve clinical outcomes in patients with AMI. Extensive investigations aimed to find and characterize novel cardioprotective strategies against MI/R have been carried out over the last three decades. Starting from the discovery of IPC [39] that provided consistent and robust results in terms of protective maneuver against MI/R injury, other conditioning approaches have been studied in experimental and clinical setting to increase the IPC clinical applicability and reduce its invasiveness. In this regard, ischemic post-conditioning (IPost) [40], an adaptive response triggered by brief episodes of I/R performed at the onset of reperfusion and the less invasive remote ischemic conditioning (RIC) [41], where brief, reversible episodes of I/R are applied in one vascular bed, tissue, or organ, have provided important insights into the field of cardioprotection. Moreover, in MI/R experimental and human models several pharmacological agents, whose use aims to activate crucial cardioprotective intracellular signaling pathways and/or limit the tissue injury, have been assessed in pharmacology-based cardioprotective protocols, that may be more suitable in the clinical setting compared with IPC [42]. However, MI/R currently lacks an effective clinical therapy; in fact, despite a large number of pre-clinical studies showing that several cardioprotective therapies were effective in reducing myocardial infarct size and improving heart function, these potential therapies showed disappointing results in the clinical evaluation. Therefore, the development of an effective cardioprotective agent to administer just before or at the onset of reperfusion still represents a major unmet medical need [3,42]. 

In the present study, we provided novel insights in the cardiac implication of endogenous SELENOT and in the IPC-like myocardial protective effects of exogenous PSELT against ex vivo induced I/R damage and ERS. We previously reported that SELENOT exerts an essential function for tissue activity and development, and is required for oxidative stress tolerance and for controlling the function of crucial organelles, such as mitochondria and ER during differentiation [16,17,43]. We also recently demonstrated that SELENOT is required during early hyperplastic growth of cardiomyocytes and is essential for cardiac differentiation and protection, likely acting as a redox-sensing protein, pointing out this selenoprotein as an emerging regulator of cardiac development and function [17]. Production and characterization of novel therapeutic peptides are under intense investigation due to their distinctive mechanism of action based on targeting specific molecules and pathways and thus, on modulating selective molecular activities, which may circumvent the typical limitations of conventional therapeutics [44]. In this regard, to overcome the potential limitations associated with the use of a full-length form of SELENOT, we designed a small peptide (PSELT) that only encompasses the redox active site (with the CVSU motif) of the protein, which is essential for its function. Accordingly, we previously reported in two different contexts the therapeutic potential of this peptide in recapitulating the activity of full-length SELENOT; in particular, we found in a mouse model of Parkinson’s disease that PSELT protects dopaminergic neurons against neurotoxin induced-oxidative stress and cell death and improves motor skills by acting at nuclear level [19]. On the other hand, the pharmacological post-conditioning with PSELT protected the rat hearts exposed to I/R injury via fundamental intracellular cardioprotective signaling mechanisms [17]. These data correlate with the crucial cytoprotective action exerted by SELENOT in brain [45], pancreas [46] and liver [47] and indicate that PSELT is able to act as a SELENOT-mimetic. These findings prompted us to further delineate the cardiac implication of SELENOT by investigating whether the pre-conditioning cardioprotective effect of PSELT results from modulating ERS and oxidative stress, as well as the endogenous SELENOT expression during I/R-induced heart dysfunction. 

### 4.1. PSELT Exerts Preconditioning-like Myocardial Protective Effects against I/R Injury and TM-Induced ERS through the Sec Residue

It has been proven that UPR signaling plays a key role in the progression of MI/R [8]; cell perturbations occurring during MI/R, such as ATP depletion, altered oxidative status and disrupted calcium homeostasis, can induce misfolded proteins accumulation in the ER lumen, which triggers UPR and ERS [48]. In this study, we established a model of ex vivo-induced MI/R by Langendorff method and examined whether ERS is functionally important for the cardioprotective role of PSELT by employing the ERS-inducing agent, TM, to enhance ERS in the IR-exposed hearts. The isolated Langendorff perfused heart model represent a useful tool for assessing the effects of pharmacological agents on heart physiology and its susceptibility to I/R insult [49]. On the other hand, TM, a naturally occurring antibiotic, blocks N-linked glycosylation by inhibiting the transfer of UDP-N-acetylglucosamine (GlcNAc) to dolichol phosphate in ER, resulting in UPR and cell death; therefore, TM is extensively used to induce ERS [50]. Our results showed that PSELT, given just before I/R induction, limited the I/R and TM-dependent contractile impairment and myocardial damage through its redox active catalytic site. In particular, the peptide markedly improved the post-ischemic contractile recovery (i.e., dLVP) without affecting the cardiac contracture, since it prevented LVEDP elevation during reperfusion, in both the absence and presence of TM; it has been reported that 4 mmHg or more above the baseline level of LVEDP represents an important index of cardiac damage in the rat heart [17,20]. The hemodynamic effect of PSELT was corroborated by its ability to mitigate the extension of myocardial infarction by reducing ischemia (with and without TM)-induced IS and LDH activity, whose release in coronary effluent reflects the severity of myocardial cellular damage during reperfusion. IS represents a crucial factor to evaluate the clinical outcomes in patients with post-ischemic heart failure and to assess the benefit of new therapies. Thus, the development of strategies that effectively decrease IS is a major objective of preclinical and clinical studies [51]. Noteworthy, we did not observe any cardioprotective action when an analogous control peptide (i.e., inert PSELT, I-PSELT), lacking the Sec residue in the active site, was infused before I/R protocol, indicating that the Sec residue within the CVSU context is responsible for the cardioprotective action of PSELT against I/R and TM. 

### 4.2. PSELT Reduces the Cardiac Expression of the ERS Markers CHOP and ATF6 and Increases Endogenous SELENOT Protein Expression 

**Figure 7:**
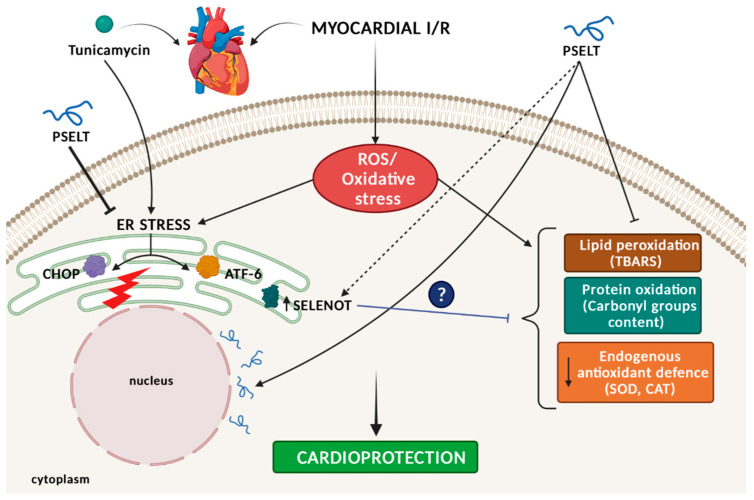
**Schematic representation of the mechanism of PSELT-induced preconditioning-like cardioprotection**. The ability of cell-penetrating PSELT to protect the heart against I/R insult, in the presence and absence of TM-induced functional ERS activation is illustrated. PSELT preconditioned the heart by (i) inhibiting lipid peroxidation and protein oxidation; (ii) enhancing SOD and CAT activities; (iii) relieving TM-induced ERS markers CHOP and ATF6. Dashed line indicates putative mechanisms, in particular related to the potential cooperative role of endogenous SELENOT in the PSELT-dependent cardioprotection. For further explanation, see text. *ATF6: activating transcription factor 6; CAT: catalase; CHOP: C/EBP homologous protein; ER: endoplasmic reticulum; ERS: endoplasmic reticulum stress; I/R: ischemia/reperfusion; ROS: reactive oxygen species; SELENOT: selenoprotein T; SOD: superoxide dismutase; TM: tunicamycin*.

Growing evidence indicates that several ERS-related pathways are involved in MI/R. Cells react to ERS by engaging the defensive process UPR; in particular, the ERS sensor transcription factor 6 (ATF6) is activated as signal transducer in UPR during MI/R injury to attempt to reduce cardiac damage [52]. In the ER cardiomyocyte, that comprises the SR/ER, ATF6 acts as a critical protein for quality control function [53]. However, when the adaptive UPR is not able to preserve ER homeostasis, a maladaptive UPR signaling shifting from pro-survival adaptive response to pro-apoptotic maladaptive response, is engaged and this process is mainly orchestrated by the homologous C/EBP protein (CHOP) [54]. The increase of this important pro-apoptotic transcription factor during ER-initiated apoptosis further contributes to cardiomyocyte death, which is the main detrimental effect of MI/R [55]. Therefore, in our study, we focused on these two ERS markers. Intriguingly, we found that PSELT reduced the cardiac overexpression of both CHOP and ATF6 induced by I/R and TM, supporting the hypothesis that the peptide can mitigate ERS response both during MI/R alone and when ERS is functionally induced by TM during MI/R. These data are consistent with the recent studies showing the key role of SELENOT in the regulation of ER proteostasis during insulin and corticotropin biosynthesis and release, in line with the identification of SELENOT as a novel subunit of the oligosaccharyl transferase (OST) complex, which contributes to hormone N-glycosylation, folding and secretion and the fact that SELENOT knockdown triggers UPR and ERS and impairs ERAD [18]. The other important mechanistic finding of our study is that PSELT infusion positively affected the cardiac expression of endogenous SELENOT during MI/R and TM; we have previously reported that SELENOT, highly expressed during cardiac embryogenesis and early development, is undetectable in mature heart, but its expression can be induced following exposure to a potent oxidative stimulus, such as I/R injury, highlighting the importance of this protein in controlling altered homoeostasis of cardiomyocytes during oxidative stress [17]. In the present study, all the hearts exposed to I/R exhibited a higher protein expression compared with sham hearts, confirming our previous observations. Interestingly, PSELT preconditioning could further enhance SELENOT expression, indicating that the peptide could stimulate the expression of the endogenous protein (Figure 7).

### 4.3. PSELT Reduces Oxidative Stress and Improves the Endogenous Antioxidant Defence System to Promote Cardioprotection

The contribution of ROS production and the resulting oxidative stress to MI/R is well established. Following MI/R, the excessive generation or accumulation of free radicals can induce macromolecule damage, including lipid peroxidation and protein oxidation, and affect the endogenous antioxidant defense, reflected by decreased activity of crucial antioxidant enzymes such as SOD and CAT, aggravating myocardial injury [56]. Importantly, compared to I/R and TM hearts, PSELT lowered TBARS levels (i.e., index of lipid peroxidation) and protein carbonyl content (i.e., index of protein carbonylation) improving myocardial SOD and CAT activities. These findings indicate that PSELT preconditioned the heart against I/R and TM damages by mitigating oxidative stress during reperfusion and boosting the endogenous antioxidant capacity. In this perspective, considering that agents able to enhance these defenses are the principal strategies underlying antioxidant therapy [57], the effect of PSELT should be regarded with interest. The antioxidant action of PSELT in the whole hearts was corroborated in vitro by evaluating the effect of the peptide against oxidative damage in cardiomyocytes. For this, we employed H_2_O_2_ to trigger oxidative stress in H9c2 cells, a cell line widely used to model cardiomyocytes in vitro given their biochemical, morphological and electrical/hormonal signaling properties [58,59]. The results show that the pre-treatment with PSELT, but not the inert form of PSELT (I-PSELT), dose-dependently counteracted the detrimental action of H_2_O_2_ on cell viability, indicating that the peptide protects cardiomyocytes from oxidative damage through Sec residue. We also found that H_2_O_2_ exposure significantly decreased SELENOT protein expression compared with control cells; interestingly, increasing concentrations of exogenous PSELT were able to rescue the protein expression with a strong stimulation at higher doses (100–1000 nM). These data suggest that SELENOT protein expression is stimulated by exogenous PSELT to protect cardiomyocytes against an acute oxidative damage, thus supporting the hypothesis that SELENOT contributes, at least in part, to the PSELT-dependent cardioprotection. To further elucidate the potential synergic action of exogenous PSELT and endogenous SELENOT in protecting cardiomyocytes from oxidative insult, we evaluated the effect of SELENOT knockdown on PSELT-induced cytoprotection in H9c2 cells during H_2_O_2_ exposure. The result indicates that SELENOT deficiency negatively affected cell viability of H9c2 with or without H_2_O_2_, indicating the importance of SELENOT in cardiomyocyte survival, and that the reduced viability after SELENOT knockdown was only partially alleviated by exogenous PSELT, reinforcing the idea that SELENOT could play a key role in the peptide antioxidant action and that the dual action of PSELT/SELENOT could be required to achieve cardioprotection. 

Confocal microscopy analysis indicates that PSELT was able to cross the cardiomyocyte plasma membrane showing a perinuclear distribution. This finding suggests that PSELT possesses an intrinsic capacity to cross the plasma membrane and that it can act intracellularly. Although further molecular and structure-activity studies are necessary to establish the mechanism of the peptide uptake and cellular compartment distribution, as well as to define the amino acids by which PSELT crosses the plasma membrane, this result correlates with our recent data according to which PSELT alone is as effective as PSELT conjugated with the cell-penetrating peptide TAT in inducing neuroprotective action [19].

## 5. Conclusions

The growing evidence regarding the key role of oxidative stress and ERS in the onset and progression of MI/R makes these processes attractive therapeutic targets for a cardiac disease that currently lacks an effective therapy. Our results overall suggest that the small mimetic PSELT (but not its inert form, I-PSELT), deriving from a protein whose genetic ablation develops a lethal phenotype and that participates to the cardiac differentiation and protection, exerts ex vivo preconditioning-like protection in hearts exposed to I/R and I/R stimulated with TM to functionally induce ERS, by mitigating cytotoxicity, oxidative stress and ERS, and by regulating the expression levels of endogenous SELENOT. The in vitro findings show that PSELT protects cardiomyocytes against H_2_O_2_ damage, and rescues the reduced SELENOT protein levels induced by H_2_O_2_ exposure, presumably acting in concert with the endogenous SELENOT to achieve cytoprotection. The ability of PSELT to penetrate the cardiomyocyte membrane suggests that the peptide acts intracellularly (Figure 7). This study further characterizes the physiological relevance of SELENOT in the heart, highlighting the possible cooperative action between PSELT and SELENOT in a physiopathological context, such as MI/R. These findings pave the way to further investigations on the mechanisms underlying the intracellular target/s of SELENOT/PSELT in the heart on the fundamental side and the potential use of PSELT in cardiac disease management on the clinical side.

## Data Availability

Data are contained within the article.

## References

[1] Virani S.S., Alonso A., Aparicio H.J., Benjamin E.J., Bittencourt M.S., Callaway C.W., Carson A.P., Chamberlain A.M., Cheng S., Delling F.N. (2021). Heart Disease and Stroke Statistics-2021 Update: A Report from the American Heart Association. Circulation.

[2] Hausenloy D.J., Yellon D.M. (2013). Myocardial ischemia-reperfusion injury: A neglected therapeutic target. J. Clin. Investig..

[3] Heusch G. (2020). Myocardial ischaemia-reperfusion injury and cardioprotection in perspective. Nat. Rev. Cardiol..

[4] Hausenloy D.J., Barrabes J.A., Bøtker H.E., Davidson S.M., Di Lisa F., Downey J., Engstrom T., Ferdinandy P., Carbrera-Fuentes H.A., Heusch G. (2016). Ischaemic conditioning and targeting reperfusion injury: A 30 year voyage of discovery. Basic Res. Cardiol..

[5] Kloner R.A., Jennings R.B. (2001). Consequences of brief ischemia: Stunning, preconditioning, and their clinical implications: Part 2. Circulation.

[6] Yellon D.M., Downey J.M. (2003). Preconditioning the myocardium: From cellular physiology to clinical cardiology. Physiol. Rev..

[7] Yellon D.M., Hausenloy D.J. (2007). Myocardial reperfusion injury. N. Engl. J. Med..

[8] Ren J., Bi Y., Sowers J.R., Hetz C., Zhang Y. (2021). Endoplasmic reticulum stress and unfolded protein response in cardiovascular diseases. Nat. Rev. Cardiol..

[9] Hetz C., Zhang K., Kaufman R.J. (2020). Mechanisms, regulation and functions of the unfolded protein response. Nat. Rev. Mol. Cell Biol..

[10] Xu C., Bailly-Maitre B., Reed J.C. (2005). Endoplasmic reticulum stress: Cell life and death decisions. J. Clin. Investig..

[11] Amen O.M., Sarker S.D., Ghildyal R., Arya A. (2019). Endoplasmic Reticulum Stress Activates Unfolded Protein Response Signaling and Mediates Inflammation, Obesity, and Cardiac Dysfunction: Therapeutic and Molecular Approach. Front. Pharmacol..

[12] Lu J., Holmgren A. (2009). Selenoproteins. J. Biol. Chem..

[13] Pitts M.W., Hoffmann P.R. (2018). Endoplasmic reticulum-resident selenoproteins as regulators of calcium signaling and homeostasis. Cell Calcium.

[14] Kalogeris T., Baines C.P., Krenz M., Korthuis R.J. (2012). Cell biology of ischemia/reperfusion injury. Int. Rev. Cell Mol. Biol..

[15] Rocca C., Pasqua T., Boukhzar L., Anouar Y., Angelone T. (2019). Progress in the emerging role of selenoproteins in cardiovascular disease: Focus on endoplasmic reticulum-resident selenoproteins. Cell. Mol. Life Sci..

[16] Anouar Y., Lihrmann I., Falluel-Morel A., Boukhzar L. (2018). Selenoprotein T is a key player in ER proteostasis, endocrine homeostasis and neuroprotection. Free Radic. Biol. Med..

[17] Rocca C., Boukhzar L., Granieri M.C., Alsharif I., Mazza R., Lefranc B., Tota B., Leprince J., Cerra M.C., Anouar Y. (2018). A selenoprotein T-derived peptide protects the heart against ischaemia/reperfusion injury through inhibition of apoptosis and oxidative stress. Acta Physiol..

[18] Hamieh A., Cartier D., Abid H., Calas A., Burel C., Bucharles C., Jehan C., Grumolato L., Landry M., Lerouge P. (2017). Selenoprotein T is a novel OST subunit that regulates UPR signaling and hormone secretion. EMBO Rep..

[19] Alsharif I., Boukhzar L., Lefranc B., Godefroy D., Aury-Landas J., Rego J.D., Rego J.D., Naudet F., Arabo A., Chagraoui A. (2021). Cell-penetrating, antioxidant SELENOT mimetic protects dopaminergic neurons and ameliorates motor dysfunction in Parkinson’s disease animal models. Redox Biol..

[20] Rocca C., Scavello F., Colombo B., Gasparri A.M., Dallatomasina A., Granieri M.C., Amelio D., Pasqua T., Cerra M.C., Tota B. (2019). Physiological levels of chromogranin A prevent doxorubicin-induced cardiotoxicity without impairing its anticancer activity. FASEB J..

[21] Penna C., Tullio F., Femminò S., Rocca C., Angelone T., Cerra M.C., Gallo M.P., Gesmundo I., Fanciulli A., Brizzi M.F. (2017). Obestatin regulates cardiovascular function and promotes cardioprotection through the nitric oxide pathway. J. Cell. Mol. Med..

[22] Rocca C., Scavello F., Granieri M.C., Pasqua T., Amodio N., Imbrogno S., Gattuso A., Mazza R., Cerra M.C., Angelone T. (2018). Phoenixin-14: Detection and novel physiological implications in cardiac modulation and cardioprotection. Cell. Mol. Life Sci..

[23] Wu H., Ye M., Yang J., Ding J., Yang J., Dong W., Wang X. (2015). Nicorandil Protects the Heart from Ischemia/Reperfusion Injury by Attenuating Endoplasmic Reticulum Response-induced Apoptosis Through PI3K/Akt Signaling Pathway. Cell. Physiol. Biochem..

[24] Morrison R.R., Jones R., Byford A.M., Stell A.R., Peart J., Headrick J.P., Matherne G.P. (2000). Transgenic overexpression of cardiac A(1) adenosine receptors mimics ischemic preconditioning. Am. J. Physiol. Heart Circ. Physiol..

[25] McQueen M.J. (1972). Optimal Assay of LDH and a-HBD at 37 °C. Ann. Clin. Biochem..

[26] Marklund S., Marklund G. (1974). Involvement of the superoxide anion radical in the autoxidation of pyrogallol and a convenient assay for superoxide dismutase. Eur. J. Biochem..

[27] Claiborne A., Greenwald R.A. (1985). Catalase activity. CRC Handbook of Methods for Oxygen Radical Research.

[28] Li T., Danelisen I., Belló-Klein A., Singal P.K. (2000). Effects of probucol on changes of antioxidant enzymes in adriamycin-induced cardiomyopathy in rats. Cardiovasc. Res..

[29] Aust S.D., Greenwald R.A. (1985). Lipid peroxidation. Handbook of Methods for Oxygen Radical Research.

[30] Reznick A.Z., Packer L. (1994). Oxidative damage to proteins: Spectrophotometric method for carbonyl assay. Methods Enzymol..

[31] Assimakopoulos S.F., Vagianos C.E., Zervoudakis G., Filos K.S., Georgiou C., Nikolopoulou V., Scopa C.D. (2004). Gut regulatory peptides bombesin and neurotensin reduce hepatic oxidative stress and histological alterations in bile duct ligated rats. Regul. Pept..

[32] Pasqua T., Rocca C., Lupi F.R., Baldino N., Amelio D., Parisi O.I., Granieri M.C., De Bartolo A., Lauria A., Dattilo M. (2020). Cardiac and Metabolic Impact of Functional Foods with Antioxidant Properties Based on Whey Derived Proteins Enriched with Hemp Seed Oil. Antioxidants.

[33] Rocca C., Grande F., Granieri M.C., Colombo B., De Bartolo A., Giordano F., Rago V., Amodio N., Tota B., Cerra M.C. (2021). The chromogranin A1-373 fragment reveals how a single change in the protein sequence exerts strong cardioregulatory effects by engaging neuropilin-1. Acta Physiol..

[34] Rocca C., De Bartolo A., Grande F., Rizzuti B., Pasqua T., Giordano F., Granieri M.C., Occhiuzzi M.A., Garofalo A., Amodio N. (2021). Cateslytin abrogates lipopolysaccharide-induced cardiomyocyte injury by reducing inflammation and oxidative stress through toll like receptor 4 interaction. Int. Immunopharmacol..

[35] Grande F., De Bartolo A., Occhiuzzi M.A., Caruso A., Rocca C., Pasqua T., Carocci A., Rago V., Angelone T., Sinicropi M.S. (2021). Carbazole and Simplified Derivatives: Novel Tools toward b-Adrenergic Receptors Targeting. Appl. Sci..

[36] Rocca C., Femminò S., Aquila G., Granieri M.C., De Francesco E.M., Pasqua T., Rigiracciolo D.C., Fortini F., Cerra M.C., Maggiolini M. (2018). Notch1 Mediates Preconditioning Protection Induced by GPER in Normotensive and Hypertensive Female Rat Hearts. Front. Physiol..

[37] Nettore I.C., Rocca C., Mancino G., Albano L., Amelio D., Grande F., Puoci F., Pasqua T., Desiderio S., Mazza R. (2019). Quercetin and its derivative Q2 modulate chromatin dynamics in adipogenesis and Q2 prevents obesity and metabolic disorders in rats. J. Nutr. Biochem..

[38] Zhang L., Liu Y., Li J.Y., Li L.Z., Zhang Y.L., Gong H.Y., Cui Y. (2018). Protective Effect of Rosamultin against H_2_O_2_-Induced Oxidative Stress and Apoptosis in H9c2 Cardiomyocytes. Oxid. Med. Cell. Longev..

[39] Murry C.E., Jennings R.B., Reimer K.A. (1986). Preconditioning with ischemia: A delay of lethal cell injury in ischemic myocardium. Circulation.

[40] Zhao Z.Q., Corvera J.S., Halkos M.E., Kerendi F., Wang N.P., Guyton R.A., Vinten-Johansen J. (2003). Inhibition of myocardial injury by ischemic postconditioning during reperfusion: Comparison with ischemic preconditioning. Am. J. Physiol. Heart Circ. Physiol..

[41] Przyklenk K., Bauer B., Ovize M., Kloner R.A., Whittaker P. (1993). Regional ischemic ‘preconditioning’ protects remote virgin myocardium from subsequent sustained coronary occlusion. Circulation.

[42] Hausenloy D.J., Garcia-Dorado D., Bøtker H.E., Davidson S.M., Downey J., Engel F.B., Jennings R., Lecour S., Leor J., Madonna R. (2017). Novel targets and future strategies for acute cardioprotection: Position Paper of the European Society of Cardiology Working Group on Cellular Biology of the Heart. Cardiovasc. Res..

[43] Pothion H., Jehan C., Tostivint H., Cartier D., Bucharles C., Falluel-Morel A., Boukhzar L., Anouar Y., Lihrmann I. (2020). Selenoprotein T: An Essential Oxidoreductase Serving as a Guardian of Endoplasmic Reticulum Homeostasis. Antioxid. Redox Signal..

[44] Heitz F., Morris M.C., Divita G. (2009). Twenty years of cell-penetrating peptides: From molecular mechanisms to therapeutics. Br. J. Pharmacol..

[45] Boukhzar L., Hamieh A., Cartier D., Tanguy Y., Alsharif I., Castex M., Arabo A., El Hajji S., Bonnet J.J., Errami M. (2016). Selenoprotein T Exerts an Essential Oxidoreductase Activity That Protects Dopaminergic Neurons in Mouse Models of Parkinson’s Disease. Antioxid. Redox Signal..

[46] Prevost G., Arabo A., Jian L., Quelennec E., Cartier D., Hassan S., Falluel-Morel A., Tanguy Y., Gargani S., Lihrmann I. (2013). The PACAP-regulated gene selenoprotein T is abundantly expressed in mouse and human β-cells and its targeted inactivation impairs glucose tolerance. Endocrinology.

[47] Tanguy Y., Falluel-Morel A., Arthaud S., Boukhzar L., Manecka D.L., Chagraoui A., Prevost G., Elias S., Dorval-Coiffec I., Lesage J. (2011). The PACAP-regulated gene selenoprotein T is highly induced in nervous, endocrine, and metabolic tissues during ontogenetic and regenerative processes. Endocrinology.

[48] Kim I., Xu W., Reed J.C. (2008). Cell death and endoplasmic reticulum stress: Disease relevance and therapeutic opportunities. Nat. Rev. Drug Discov..

[49] Bell R.M., Mocanu M.M., Yellon D.M. (2011). Retrograde heart perfusion: The Langendorff technique of isolated heart perfusion. J. Mol. Cell. Cardiol..

[50] Hakulinen J.K., Hering J., Brändén G., Chen H., Snijder A., Ek M., Johansson P. (2017). MraY-antibiotic complex reveals details of tunicamycin mode of action. Nat. Chem. Biol..

[51] Gibbons R.J., Valeti U.S., Araoz P.A., Jaffe A.S. (2004). The quantification of infarct size. J. Am. Coll. Cardiol..

[52] Doroudgar S., Thuerauf D.J., Marcinko M.C., Belmont P.J., Glembotski C.C. (2009). Ischemia activates the ATF6 branch of the endoplasmic reticulum stress response. J. Biol. Chem..

[53] Glembotski C.C. (2014). Roles for ATF6 and the sarco/endoplasmic reticulum protein quality control system in the heart. J. Mol. Cell. Cardiol..

[54] Szegezdi E., Logue S.E., Gorman A.M., Samali A. (2006). Mediators of endoplasmic reticulum stress-induced apoptosis. EMBO Rep..

[55] Glembotski C.C. (2007). Endoplasmic reticulum stress in the heart. Circ. Res..

[56] Kurian G.A., Rajagopal R., Vedantham S., Rajesh M. (2016). The Role of Oxidative Stress in Myocardial Ischemia and Reperfusion Injury and Remodeling: Revisited. Oxid. Med. Cell. Longev..

[57] Forman H.J., Zhang H. (2021). Targeting oxidative stress in disease: Promise and limitations of antioxidant therapy. Nat. Rev. Drug Discov..

[58] Hescheler J., Meyer R., Plant S., Krautwurst D., Rosenthal W., Schultz G. (1991). Morphological, biochemical, and electrophysiological characterization of a clonal cell (H9c2) line from rat heart. Circ. Res..

[59] Branco A.F., Pereira S.P., Gonzalez S., Gusev O., Rizvanov A.A., Oliveira P.J. (2015). Gene Expression Profiling of H9c2 Myoblast Differentiation towards a Cardiac-Like Phenotype. PLoS ONE.

